# The Vascular Niche for Adult Cardiac Progenitor Cells

**DOI:** 10.3390/antiox11050882

**Published:** 2022-04-29

**Authors:** Diego Herrero, Guillermo Albericio, Marina Higuera, María Herranz-López, Miguel A. García-Brenes, Alejandra Cordero, Enrique Roche, Pilar Sepúlveda, Carmen Mora, Antonio Bernad

**Affiliations:** 1 Department of Immunology & Oncology, National Center for Biotechnology (CNB-CSIC), Campus de Cantoblanco de la Universidad Autónoma de Madrid, 28049 Madrid, Spain; diego.herrero@correo.gob.es (D.H.); galbericio@cnb.csic.es (G.A.); mhiguera@cnb.csic.es (M.H.); ma.garcia@cnb.csic.es (M.A.G.-B.); acordero@cnb.csic.es (A.C.); cmora@cnb.csis.es (C.M.); 2 Institute of Research, Development and Innovation in Health Biotechnology of Elche (IDiBE), Universitas Miguel Hernández (UMH), 03202 Elche, Spain; mherranz@umh.es; 3 Institute of Bioengineering and Department of Applied Biology-Nutrition, Miguel Hernández University, 03202 Elche, Spain; eroche@umh.es; 4 Instituto de Investigación Sanitaria y Biomédica de Alicante (ISABIAL), 03010 Alicante, Spain; 5 Regenerative Medicine and Heart Transplantation Unit, Instituto de Investigación Sanitaria La Fe, Avda, Fernando Abril Martorell 106, 46026 Valencia, Spain; pilar.sepulveda@uv.es

**Keywords:** heart, cardiac turnover, endothelial cell, cardiac progenitor, stem cell, niche, vascular niche, Bmi1, polycomb, ROS

## Abstract

Research on cardiac progenitor cell populations has generated expectations about their potential for cardiac regeneration capacity after acute myocardial infarction and during physiological aging; however, the endogenous capacity of the adult mammalian heart is limited. The modest efficacy of exogenous cell-based treatments can guide the development of new approaches that, alone or in combination, can be applied to boost clinical efficacy. The identification and manipulation of the adult stem cell environment, termed niche, will be critical for providing new evidence on adult stem cell populations and improving stem-cell-based therapies. Here, we review and discuss the state of our understanding of the interaction of adult cardiac progenitor cells with other cardiac cell populations, with a focus on the description of the B-CPC progenitor population (Bmi1+ cardiac progenitor cell), which is a strong candidate progenitor for all main cardiac cell lineages, both in the steady state and after cardiac damage. The set of all interactions should be able to define the vascular cardiac stem cell niche, which is associated with low oxidative stress domains in vasculature, and whose manipulation would offer new hope in the cardiac regeneration field.

## 1. Introduction

Tissue homeostasis is guaranteed by the highly regulated activity of adult stem cells (ASC) and progenitors, contributing also in damage responses [1,2]. In the best characterized ASC models, stem cell niches have been defined that critically regulate stem cell function. Currently, the combination of a stem cell niche with the specific ASC populations is recognized as the functional unit for maintenance and regulation of ASC [3]. Niches function both though direct cell–cell contact and by releasing soluble factors that modify the environment of the engrafted ASC [4].

Although ASC niches have been defined in most adult stem cell linages, the hematopoietic stem cell (HSC) niche is the best understood [4], and the cardiac progenitor cell niche, as well as the proper cardiac progenitors, are less clearly defined. In this review, we compare the evidence accumulated on the best-characterized models (i.e., HSC, neural stem cells, NSC, muscular skeletal stem cells, and satellite cells) [4,5,6] with those related with cardiac cell turnover, as well as the different proposals for cardiac niches and progenitor interactions. Finally, all this previous information is discussed in relation to the knowledge accumulated for the Bmi1^+^-expressing cardiac progenitor cell (Bmi1-CPC: B-CPC) population. B-CPC [7] is a resident multipotent population that mainly is associated with cardiac vessels; those B-CPC closer to vasculature are maintained in quiescence in low reactive oxygen species (ROS) domains. B-CPC location and proliferative activation is highly modulated by ROS levels, associated with several forms of damage, including myocardial infarct. All results clearly suggest a vascular cardiac niche, at least for B-CPC, and indicate that this line of research could help to define the mechanism associated with cardiac turnover in mammals.

## 2. The Adult Progenitor Cell Niche

Tissue homeostasis, repair, and damage response relies on the regulated activity of tissue-specific adult stem cells (ASCs)/progenitors [1,2]. The stem cell niche, defined as the local (micro)environment surrounding a stem cell, is now recognized as the functional unit for ASC maintenance and regulation. Stem cell niches are dynamic functional domains that influence and condition ASC behavior to govern tissue homeostasis under diverse physiological (development and aging) and pathological conditions [3]. The stem cell niche can be co-opted [8,9,10] in human pathologies (cancer and other chronic diseases).

**Figure 1:**
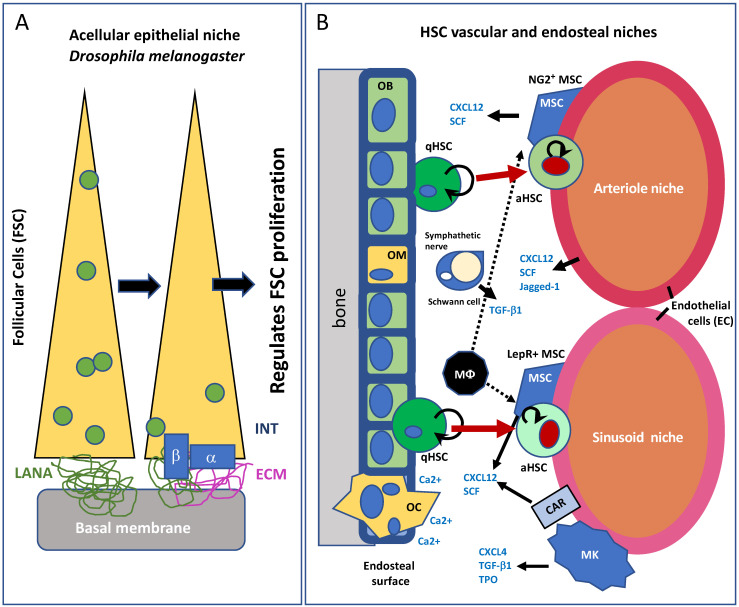
Examples of stem cell niches. (**A**) The acellular epithelial niche in *Drosophila melanogaster*. The ovary follicular cell compartment (FSC) is indicated with a yellow square; INT, integrins; α/β subunits; ECM, extracellular matrix; LANA, laminin A. Adapted from O’Reilly et al. (2008) [15]. The model is not conserved in mammals. (**B**) Simplified scheme for the bone marrow hematopoietic stem cell (HSC), composed of two subniches, the endosteal and the vascular niches. qHSC, quiescent hematopoietic stem cells; aHSC, activated hematopoietic stem cells; MSC, mesenchymal stem cells; MΦ, macrophages; MK, megakaryocytes; OB, osteoblasts; OC, osteoclasts; OM, osteomacs; CAR, CXCL12-abundant reticular cells.

The niche concept was first proposed in 1978 by Ray Schofield for hematopoietic stem cells (HSC), referring to supporting cells and soluble factors that influence HSC behavior [11]. This concept has been further demonstrated in most of the ASC populations studied [12]. Currently, on the basis of its structure, we can distinguish between the epithelial crypt (e.g., intestinal stem cells and hair follicle stem cells) (Figure 1A) and the perivascular niche (e.g., hematopoietic stem cells, neural stem cells, cancer stem cells) (Figure 1B). The use of novel experimental models with intrinsic simplicity (*D. melanogaster* or *Caenorhabditis elegans*, etc.) have been instrumental in defining the plasticity of stem cell niches [13]. For example, it has recently been revealed that two stem cell niches contribute to controlling *Drosophila* blood cell homeostasis: In the lymph gland, the niche called posterior signaling center controls only a subset of the heterogeneous hematopoietic progenitor population, and the vascular system acts as a second niche to control lymph gland homeostasis, activating the fibroblast growth factor (FGF) pathway in hematopoietic progenitors to preserve progenitor pools and prevent blood cell differentiation [14].

ASC niches function through direct cell–cell contact and by releasing soluble factors that modify the environment of the engrafted ASC. However, recent studies [4,16,17,18,19,20] on the HSC niche, undoubtedly the best-studied model of the ASC niche, have uncovered new layers of regulatory complexity (Figure 1). For instance, HSC progeny themselves regulate HSC behavior, lineage-biased differentiation of HSC is regulated by distinct niches, and HSC can remodel their perivascular niche [4,17,18]. This high level of complexity is evident in other well-described ASC niches. For example, several signaling pathways regulate neural stem cells (NSC) through both direct cell–cell contact and soluble factors secreted by endothelial-related cells [5,18]. Likewise, several cell types regulate intestinal- and follicle-stem cell behavior, including bone marrow (BM)-derived cells [19,20] (Figure 1). Therefore, it is expected that any ASC described could be organized similarly with specific niches that would adjusted to the tissue requirement.

## 3. Translational Relevance of the Stem Cell Niche

From a therapeutic perspective, the most efficient regenerative therapies will likely focus on a combination of stem cell therapy and simultaneous targeting of the endogenous stem cell niche. In the context of bone marrow (BM) transplantation, myeloablative regimens are commonly required to obtain substantial engraftment of cells, and it has been shown in mice that much higher doses of transplanted BM cells are required when myeloablation is omitted [21]. However, conventional myeloablation cannot be considered in some human pathologies due to anticipated high-level toxicity, for example in patients with Fanconi anemia (FA) (DNA repair phenotype), that, in addition, do not obtain good results in allogeneic transplant. In this case, the use of autologous corrected HSC (lentiviral-mediated gene therapy) populations resulted in a good level of engraftment in a clinical trial (NCT03157804). The authors of this study [22] demonstrated that gene-corrected hematopoietic cell therapy reproducibly confers both engraftment and proliferation advantages. The significant engraftment of corrected HSC in non-conditioned patients with FA strongly support the novel notion that gene therapy is a low-toxicity therapeutic option for this disorder, where BM failure is the primary pathological feature and, likely, endogenous FA-HSC (and perhaps some other niche elements) could be chronically damaged and be out-competed by transplanted autologous lentiviral-cured HSC. Finally, it must be considered that due to the dynamics of human BM, 1–2 years are typically needed to generate a sufficient number of corrected mature cells derived from the long-term HSC post-transplantation [23], which is equivalent to 2–3 months in the mouse model. This strong difference in the physiology and regulation in BM could also be of interest for other human cell therapy approaches.

BM transplantation has been the origin of many concepts in regenerative medicine and will likely aid in resolving the critical issues that need to be addressed for efficient cell therapy in solid organs. The above example would suggest the requirement for available niches to improve the engraftment of any transplanted ASC, including cardiac progenitor cells.

## 4. Cardiac Cell Turnover: A Critical Overview

### 4.1. Cardiac Stem/Progenitor Cell Identification and Its Caveats

The concept of the mammalian adult heart as a terminally differentiated organ has been challenged in recent years by several detailed and elegant studies from independent laboratories. While various reports describe that mature cell lineages and adult progenitors contribute to cardiac cell turnover, the source(s) of the new cells in the adult mammalian heart remain enigmatic [24]. Classical studies on ASC definition and characterization have relied on the efficient and specific ASC labeling and tracing of their progeny, which has helped to define several niches for candidate adult cardiac stem cells (CSC) and to solidify the concept that the binomial stem cell niche is the actual functional unit [3].

The unique physiology and structure of the heart, however, renders these strategies less useful for the identification of cardiac cells responsible for normal heart normal turnover (probably some cardiac stem/progenitor cell populations) and their putative niches. Some of the main issues are: (1) The adult heart has a very low cell turnover, especially for the cardiomyocyte lineage (<3% per year); therefore, adult CSC are not the unique quiescent cells in the heart, and the biological effects of adult CSC (scarce cell progeny) take long periods of time to play out. (2) The absence of a robust cell turnover dynamic based on a CSC population and an efficient organ regeneration capacity points to (probably an artificial enrichment) trans-differentiation and fusion events, despite the fact that their manifestation may not be more frequent than in other adult tissues. (3) Until recently, cell lineage tracing studies were based only on one genetic locus, and no one protein has been described as necessary and sufficient to identify CSC. (4) Remarkable differences between in vitro and in vivo behavior and cell differentiation potential of cardiac cells have been reported, which are similar to findings in other tissues/lineages (e.g., pericytes). (5) The majority (>90%) of murine cardiomyocytes are binucleated, and classical proliferation assays do not differentiate between proliferation and polynucleation of cardiomyocytes; moreover, the functional relevance of that polynucleation is unknown. These issues have wrapped the investigation of adult cardiac progenitor cells and cardiac regeneration in a complex network of likely incompatible concepts.

Cardiomyocyte proliferation is a regulated process in pig hearts [25] and in young human individuals (<20 years old) [26]; cardiac proliferation is enhanced in myocardial infarction (MI) [27]. All these results suggest the contribution of an atypical resident progenitor/stem cell population from which new myocytes can arise, as has recently been established for smooth muscle cells (SMC) in mammalian arteries [28]. An alternative proposal, however, is the de-differentiation/proliferation/re-differentiation of mature cardiomyocyte populations [29,30], which still awaits robust empirical verification. Moreover, both processes could be acting coordinately.

**Figure 2:**
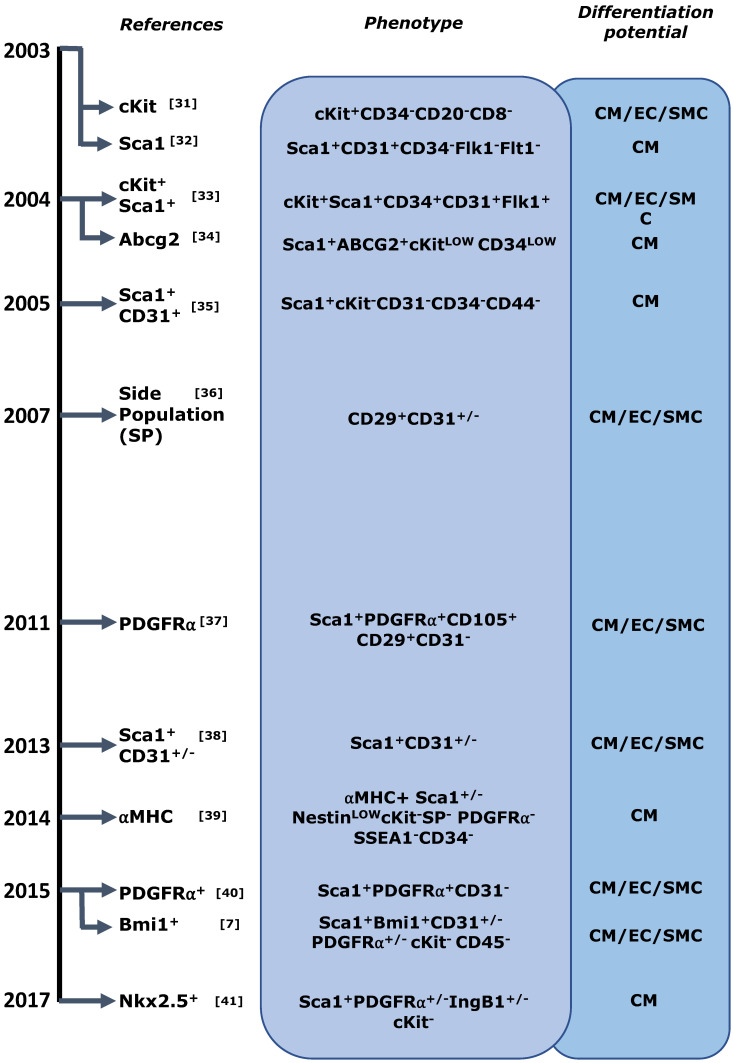
Description of main candidate cardiac stem/progenitors [31,32,33,34,35,36,37,38,39,40,41].

Beltrami et al. [31] staked the first claim on a CSC population through the identification of cKit^+^ cells, and several other progenitor-like cell populations have been described in the ensuing 20 years (Figure 2). 

Most of these proposals are based on cell-surface proteins that are also expressed on other ASC (including c-Kit, Sca1, and Abcg2 (side population)), markers associated with general or cardiac progenitor function (such as Isl1 or Bmi1), or on standard lineage tracing experiments using one definitive gene [7,42]. Only one study analyzed the functional consequences of conditional ablation and highlighted both their non-essential role in homeostasis and their physiological relevance in neovascularization after MI [43].

Although several markers have been proposed for the identification/isolation of cardiac resident stem/progenitor cells (multipotent cells) from the heart (Figure 2), c-Kit^+^ cardiac cells were the first and most intensively studied cell population [44] as a potential resource for cardiovascular therapy; they were given the descriptive name of cardiac stem cells (CSC). However, later findings from basic and preclinical research, together with the failure of several clinical trial evaluation, have fueled a long debate over the existence of c-Kit^+^ CSC that has led to a general skepticism in the field [45].

Although the function of endogenous c-Kit^+^ cells is still far from being completely dilucidated [46], several relevant clues have been reported, indicating that some technical limitations could be playing a significant role at the origin of the described differences [47,48,49]. Hence there is no globally accepted mechanism (or associated niches) of endogenous c-Kit^+^ cells. Recently, it has been demonstrated that the functional benefits of cell therapy with exogenous c-Kit^+^ cells are associated to acute inflammatory-based wound healing responses, involving macrophage populations that regulate cardiac fibroblast activity [50]. In any case, multiple questions remain open to reconcile all published results.

In this sense, it has been proposed that the number of cardiac c-Kit^+^ cells with clonogenic potential in the mouse adult heart is low (≈10% of total c-Kit^+^ cells) [48] and that the *c-Kit* expression level is much lower in c-Kit^+low^CD45^-^CD31^-^ cells than in other known cardiac c-Kit^+^ cell lineages [47]. Indeed, the differentiation potential of true multipotent CSC is only associated with ≈1% of clonogenic cells [48]. 

Scarce research has been invested in defining the potential niches for c-Kit^+^ CSC, but prior data indicated that two distinct subpopulations of endogenous CSC can be identified in adult human tissue: myogenic CSC (mCSC), which are characterized by the presence of c-Kit receptor and localization in a myocyte niche surrounded by mature cardiomyocytes, and vasculogenic CSC (vCSCs), which express c-kit and kinase insert domain receptor (KDR) and associate with a putative vascular niche close to the wall of blood vessels. Both were described as stem cell-like populations, with self-renewing, clonogenic, and multipotent capacities [51]. A better comprehension of the localization and regulation of the cardiac niche will be key for resolving the uncertainties in the involvement of cKit^+^ cardiac progenitor cells/stem cells on heart homeostasis and damage repair.

### 4.2. Progenitor Cell Interactions in the Adult Heart and Alternative Sources of Heart Turnover

The majority of the reports on the interactions of cardiac progenitors with their environment are derived from in vitro experiments, and outcomes of crosstalk have mainly been focused on paracrine effects. Some of the first studies showed that, in vitro, mature cardiomyocytes are connected to cardiac progenitor cells through focal adhesions such as integrin-β1 [52]. This cell–cell interaction was believed to favor cardiac progenitor differentiation, which could be directed by TGF-β1 signaling [53]. Accordingly, co-culture with mature cardiomyocytes was the most efficient differentiation protocol to obtain cardiac progenitor-derived cardiomyocytes [54]. Previous results also indicated a paracrine effect of peri-infarcted tissue by the elevated expression of stromal cell-derived factor 1 alpha (SDF1α), a powerful chemokine that recruited ckit+ cardiac stem cells [55].

Concurrently, several groups focused on the regulation of cardiac progenitor cell behavior by cardiac fibroblasts and epicardial-derived cells; co-culture of epicardial-derived cells with cardiac progenitors induces the expression of metalloproteinases and favors proliferation of cardiac progenitors [56], and fibroblast-conditioned medium also induced cardiac progenitor differentiation through the Wnt pathway. This effect may be driven by several chemokines, as demonstrated for fibroblast growth factor 2 (FGF-2) and insulin-like growth factor 1 (IGF-1) [57]. In addition, fibroblasts were found to regulate cardiac progenitor behavior through the production of extracellular matrix, which is composed of several proteins including collagen types I, III, and V and laminin. Decellularized human heart extracellular matrix scaffolds (dECM), which retain three-dimensional architecture and vascularity, were used to promote cardiac differentiation pathways both in human and mouse cardiac progenitors [58] and, moreover, the extracellular matrix composition was demonstrated to regulate cell cycle status [59].

Endothelial cells have also been suggested to regulate cardiac progenitor cell behavior through both by secreted factors and cell–cell contact.

Notch receptors are highly expressed by endothelial cells, and Notch signaling plays several roles in regulating stem cell self-renewal decisions. Notch signaling was found to be necessary for correct cardiac progenitor cell expansion and differentiation by inducing the expression of cardiomyogenic-related genes such as *Nkx2.5* [60]. In the adult mouse heart, the abundance of Sca1^+^ cardiac progenitor cells was reported to increase after activation of Notch signaling, and the majority of c-Kit^+^ and Sca1^+^ cardiac progenitor cells express the Notch1 receptor. Endothelial cells also have instructive roles through paracrine signaling. SDF-1a is a chemokine expressed by several cell types including endothelial cells. In the heart, SDF-1a is linked to c-Kit^+^ progenitor cell distribution and to the motility of cardiac progenitors [55]. Some CSC populations have been also shown to express vascular endothelial growth factor receptor 1 and 2, and a role for VEGFA has been proposed in adhesion and migration of cardiac progenitor cells [61].

The regional hypoxia that accompanies acute MI induces in vivo cardiac progenitor cell proliferation [42,43]. Accordingly, oxygen tension (pO_2_) affects cardiac progenitor cell behavior in vitro. Cardiac progenitors cultured in low oxygen (<3%) upregulate progenitor-related markers such as c-KIT and BMI1 [62]. In addition, low oxygen also improves the survival, cardiac healing, cell migration, genomic stability, and vasculogenic potential of cardiac progenitor cells [63].

Finally, in addition to the activity of resident cardiac progenitor cells, mesenchymal-to-endothelial transition, BM-derived circulating endothelial progenitor cells, and proliferation of pre-existing endothelial cells have all been reported to contribute to cardiac neovascularization [64]. These factors/parameters also likely contribute to endothelial turnover in murine models. We found a similar scenario in our B-CPC model for cardiac fibroblasts: progenitor cells (Gli1^+^) [65], endothelial-to-mesenchymal transition, and pre-existing fibroblasts are reported to contribute to cardiac fibrosis after acute MI [66]. The cardiomyocyte turnover rate in an adult heart (<3% of total cardiomyocytes/year) could be dependent on resident cardiac progenitor cells, endothelial-to-cardiomyocyte trans-differentiation (not yet strongly confirmed) [67], and/or on the proliferation of pre-existing cardiomyocytes [29,30].

## 5. The Cardiac Stem Cell Niche in the Adult Heart

The mesothelial sheet of cells that cover the heart, called the epicardium, was the first proposed niche-like structure in the developing and adult heart. These cells, which express the transcription factor Wilm’s tumor-1 (WT1), contribute to cardiomyocytes, endothelial cells, and SMC during cardiac development. In adulthood, re-expression of WT1 was reported to generate cardiomyocytes de novo through priming by the pleiotropic factor thymosin β4 [68].

The subepicardium, the cell layer below the epicardium, was also proposed as a microenvironment for adult cardiac progenitor cells, as it has the lowest capillary density of the ventricular myocardium and, therefore, is relatively hypoxic. Hypoxic cardiac cells preferentially located in the subepicardium (hypoxia-inducible factor 1-alpha-positive (Hif1α^+^)) have a metabolic footprint similar to that of other tissue-specific stem cells [69]. The cardiac progenitor cell niches, however, are not restricted to the epicardium. cKit^+^ cardiac progenitors were reported in hypoxic niches throughout the myocardium, mainly in the atria and in the apex due to the low hemodynamic stress [70,71]. While the majority of the studies show no spatial distribution of putative cardiac progenitor cells, there is a general consensus on the characteristics of their niche: low oxidative damage and low oxygen levels.

More recently, the search for cardiac progenitor cell niches has focused on the vasculature. An endothelial fate-mapping study showed that mature endothelial cells trans-differentiate to cardiomyocytes in the murine adult heart, although the endothelial–mesenchymal transition was not restricted to the cardiomyocyte lineage [67]. A study by Chen et al. showed that mature endothelial cells trans-differentiate to pericytes and vascular SMC in the adult heart [72]. In relation to cardiac progenitor cell location, the aforementioned study by Fioret et al. suggested that >70% of perivascular Sca1^+^ progenitor cells are derived from endothelial-related cells [67]. These perivascular Sca1^+^ cells were in a quiescent state and expressed early cardiogenic markers. Cell distribution analyses showed that the majority of endothelial-derived cells were located in the right ventricle and in the lateral free wall of the left ventricle, in both cases, close to the epicardium and to the right and circumflex coronary arteries [67]. Some of these findings are partially conflictive because many of the driver sequences used in the lineage tracing analyses and assumed to be specific for endothelial cells are expressed by B-CPC cardiac progenitors at relevant levels [73]. On the basis of the demonstrated role in other adult niches, both pericytes and telocytes could be considered as plausible niche components. In the adult bone marrow, some pericyte subsets have been demonstrated to be involved hematopoietic support, regulating HSC behavior, maintenance, quiescence, and trafficking through paracrine effects [74]. Concerning telocytes, although they are still a controversial cell type characterized by the presence of a particular kind of prolongation, known as telopodes, that typically organize in intricate networks within the stromal space of most organs, they have been proposed to form part of most adult stem cell niches [75].

Although the perivascular location of cardiac progenitors may seem contradictory to the concept of a hypoxic niche, in vivo measurement of pO_2_ in the BM confirmed that it is a hypoxic tissue despite its high vascularity [76]. The authors found that pO_2_ decreases in large-sized vessel perivascular areas, which is contrary to the idea that small-sized vessel perivascular areas are the most hypoxic. The authors suggested that oxygen consumption by the cells of the BM niche might be more relevant than the distance to the vasculature [77]. 

## 6. The Bmi1+ Cardiac Progenitor Cell Niche in the Adult Heart

The BMI1 protein, forming part of the polycomb repressive complexes 1 (PRC1), is a transcriptional suppressor that contributes to maintain the self-renewal and proliferation of many tissue-specific stem cells [73]. Numerous studies have shown that BMI1 serves as a key regulator in some tumorigenic pathways. Our understanding, however, of BMI1 in terminally differentiated organs, such as the heart, is relatively limited [78]. Interestingly, in the context of its regulatory potential, BMI1 has been demonstrated as negative factor for direct cardiac reprograming [79]. As a differential strategy to define elusive cardiac stem cells, expression of BMI1, as the most representative marker of mouse adult stem cell systems, was evaluated in the adult mouse heart (see below).

### 6.1. Adult Cardiac Multipotent Progenitor Cells Are Characterized by the High Expression of Bmi1

**Figure 3:**
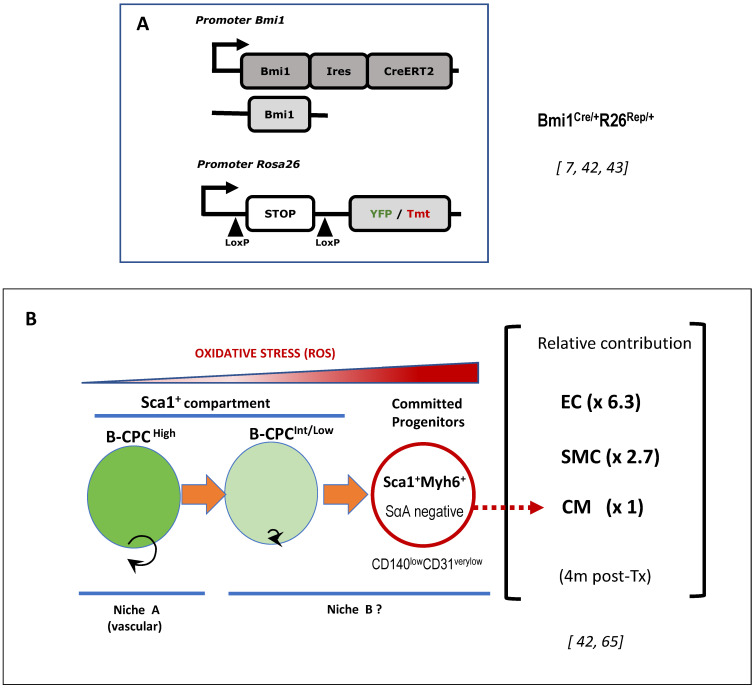
Working model for the differentiation of B-CPC driven by ROS levels. (**A**) Genetic scheme of the mouse strain Bmi1Cre/+R26Rep/+ used for the characterization of B-CPC regulation/differentiation. Rep, indicates the use of YFP or Tmt as reporter; LoxP, specific LoxP sites for the Cre recombinase. (**B**) The B-CPC compartment is not in equilibrium with exogenous or endogenous precursors. B-CPC is a heterogeneous Sca1+ population with respect to Bmi1 expression levels. B-CPChigh correspond to the more immature and quiescent progenitors and most probably associate with a dedicated vascular niche (Niche A). It is proposed that later, ontogenically speaking, subpopulations B-CPCint/low and Sca1+Myh6+ cardiomyocyte (CM) committed progenitors could be associated with other pro-differentiation-biased niches (Niche B). In fact, it has been demonstrated that proliferative activated B-CPC are located more distantly with respect to the cardiac vessels. Progeny of B-CPC, 4-months after labeling is composed (relative to CM) by a 2.7 fold increase for smooth muscle cells (SMC) and 6.3-fold for endothelial cells (EC).

Using the Bmi (CreER/^+^), the Rosa26 (YFP/^+^) (Bmi1-YFP) mouse model [80], which allows the inducible labeling (YFP) of Bmi1^+^ cells by tamoxifen (Tx) administration, and alternativelly the Bmi1^(GFP/+)^ (Bmi1-GFP) knock-in transgenic strain [7] (Figure 3A), we demonstrated the existence of a Bmi1^+^ heart population (as well their progeny) some years ago [7]. The Bmi1^+^-expressing cardiac progenitor cell (Bmi1-CPC: B-CPC) population was defined by FACS of the non-cardiomyocyte population as approximately 75,000 B-CPC per adult heart. In addition, it was demonstrated that B-CPC were able to differentiate in vitro to the three main cardiac lineages. Pulse-chase analysis showed that the B-CPC population remained relatively stable for extended periods, which suggests that this labeled Bmi1^+^ population could contain cardiac progenitors with substantial self-maintenance potential (up to 1 year), and that pulse-labelled cells are not displaced by subsequent proliferation/differentiation waves. Similar results were independently reported in Bmi-1 GFP^hi^ heart cells [81,82]. 

B-CPC is a heterogeneous population, negative for pericyte-related α-smooth muscle actin (αSMA), pericyte/endothelial melanoma cell adhesion molecule (CD146), and fibroblast-related markers CD90 and FSP-1. They are also negative for the mesenchymal-related markers nestin and endoglin (CD105). However, they contain two major, and mutually exclusive, subpopulations: B-CPC-PDGFRα^high+^CD31^neg^, which express high levels of platelet-derived growth factor receptor alpha (PDGFRα), and a B-CPC-CD31^+^ PDGFRα^neg^ population. It was previously suggested that PDGFRα expression is associated with the clonogenic capacity of Sca1^+^ cardiac progenitor cells [40]. Comparative analysis of the in vitro differentiation profiles strongly suggested that the B-CPC-CD31^+^ PDGFRα^neg^ subpopulation is the likely main contributor to the endothelial progeny [42].

B-CPC showed a broad distribution by immunostaining in Bmi1-YFP heart serial sections, 5 days after Tx induction; B-CPC were detected in small-sized clusters that were mainly localized in two locations: a minority in clear association with muscle fibers and, the majority, in association with heart vessels; no YFP^+^ cardiomyocytes were detected at this time after Tx induction. YFP^+^ cardiomyocytes emerged at 2 to 12 months after Tx induction. B-CPC also contributed to endothelial and smooth muscle lineages in vivo. These results demonstrate that high levels of Bmi1expression identify a non-cardiomyocyte resident cardiac population that contributes, in a steady state, to the main lineages of the heart in vitro and in vivo [7,40]. Finally, clonal in vivo dilution assays demonstrated that at 4 months after Tx induction, in homeostasis, labeled cells formed small clusters (2–5 cells) containing mature cells of the main heart lineages, with a bias for the endothelial lineage (>63%), and most of the clusters contained mature cells belonging to one or two lineages, with a minority (2–5%) showing tri-lineage differentiation [42] (Figure 3B). B-CPC differentiation is enhanced by oxidative stress (see Section 6.2).

In response to severe damage (i.e., acute myocardial infarct), lineage-tracing studies showed that B-CPC became proliferatively activated [42,73], even incrementing the B-CPC numbers during the first days [73,82]; moreover, its progeny showed an enhanced contribution to de novo cardiomyocytes (YFP+ CM) compared to age-paired non-infarcted adult hearts. Equivalent effects were found after whole-body γ-irradiation (γ-IR; 9 Gy) and paraquat (PQ) treatment [42]. Summing up, the B-CPC population seems to be quite resistant to several forms of oxidative stress and, on the contrary, becoming activated shortly after damage and incrementing their contribution to all mature cardiac lineages, although with different efficiencies [42].

The physiological role of B-CPC has been validated by genetic ablation using the expression of DTA coupled to Bmi1 regulatory sequences. In homeostasis, a substantial depletion (>92%) of the Bmi1^+^ cell population did not cause any evident phenotype, including no major differences in conventional echocardiography parameters or in cardiac histology, even in one-year-old mice, and cells showed a substantial recovery 4 months after DTA. These results suggest that, in homeostasis, B-CPC are not essential for cardiac function and that the heart has compensatory mechanisms to replenish this cardiac progenitor population [43]. In sharp contrast, when B-CPC ablation was coupled to acute MI, animals manifested signs of cardiac dysfunction 2 months post-ablation, which progressed over the next 2 months, affecting the survival of B-CPC-depleted mice. Histological analysis of B-CPC-depleted hearts showed eccentric cardiac hypertrophy (an ischemic-dilated cardiac-like phenotype) characterized by left ventricular chamber dilatation, thinning of the interseptal wall, and reduced ejection fraction. No significant differences were found for fibrosis, although there was a clear increase in left ventricular scar size in B-CPC-depleted hearts. More importantly, histology, FACS, and RT-qPCR analyses confirmed that the significant deficit in B-CPC changed the angiogenic response to acute MI. It was estimated that at 4 months after MI, the B-CPC population contributed up to 20% of total endothelial cells in the infarcted heart [43]. In conclusion, the B-CPC population seems to be relevant to preventing dysfunctional ventricular remodeling and cardiac failure after acute MI.

Globally, the results for B-CPC resemble those described for the recently defined arterial Sca1^+^ vascular stem cells (Sca1^+^ VSCs) [28]. Sca1^+^ VSCs only contribute to the de novo generation of one cell lineage, SMC, and only in response to severe damage. Genetic lineage tracing using dual recombinases identified a Sca1^+^PDGFRa^+^ VSC subpopulation as being responsible for SMC generation, and the genetic ablation of Sca1^+^ VSCs significantly impaired artery repair. These results provide genetic evidence of a bona fide atypical (monopotential) Sca1^+^ VSC population that specifically produces SMC with a relevant role in vessel repair only after severe injury [28]. In steady-state conditions, B-CPC likely contribute to minimizing the physiological and age-related wear and tear, whereas upon increased levels of stress/damage (progressively increased with aging), they are overactivated to counter the depletion of mature cells.

### 6.2. Oxidative Stress Plays a Central Role in B-CPC Regulation

The relationship between ASC and reactive oxygen species (ROS) has been studied extensively in several ASC populations [57,83], but the role of ROS in adult cardiac cell turnover and progenitor behavior has been poorly explored. Many ASC are associated with non-hypoxic adult vasculature [84,85], and the adult heart has the highest pO_2_ of any organ. Several attempts have been made to associate putative cardiac stem cells with the lowest endogenous ROS levels, on the basis of expression of the hypoxic marker HIF-1α. Recent reports identified rare proliferative HIF-1α^+^ cardiomyocytes [86] and hypoxic cells associated with the epicardium, the least-vascularized heart area [87].

**Figure 4:**
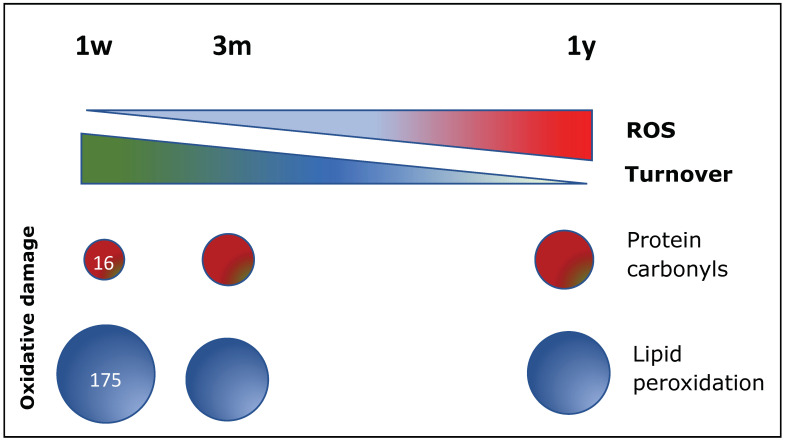
Oxidative stress is a driving force for homeostatic turnover in mouse heart. Total levels of reactive oxygen species (ROS) and regeneration capacity (turnover) are compared in mice aged 1 week old, 3 months old, and 1 year old. Levels of molecular damage (protein carbonyls and lipid peroxidation (malondialdehyde, MDA) were evaluated at those ages. Numbers (1 week) correspond to the levels (μM) of carbonyls and MDA; the surface of the indicated circles are proportional to the quantitative evaluation.

In the murine heart, we found a progressive increase in total ROS levels with age that was associated with decline in the B-CPC population and the heart turnover (Figure 4), although not in perivascular areas [65]; concerning ROS-derived damages, an increment in protein carbonyl damage is maintained along the whole life, whereas lipid peroxidation (malondialdehyde; MDA), globally higher, is only significative higher during the first weeks of life (Figure 4).

Genetic manipulation of ROS levels confirmed the importance of perivascular ROS for B-CPC homeostasis. The global decrease in oxidative damage in transgenic glucose-6-phosphate dehydrogenase (G6PD^Tg^) mice, which have low levels of oxidative stress in several organs, including the heart, and an extended lifespan [88], promoted a shift in the cardiac tissue composition resembling that of a younger animal, with high levels of B-CPC and delayed B-CPC age-associated loss. Conversely, the increase in vascular oxidative damage in *Sod3*-knockout mice [89] accelerated the age-related loss of B-CPC [65].

ROS have important functions in adult tissue homeostasis and affect both differentiated cells and ASC [90]. Low ROS levels are relevant for ASC proliferation, and elevated ROS production primes progenitor differentiation in several tissues [83,91]. The heart is no exception, and its highest regeneration capacity is found during development, which resides in a relatively hypoxic environment [92]. These arguments strongly suggest a role for oxidative stress in adult cardiac progenitor cell behavior. In this context, *Bmi1* overexpressed by B-CPC works as an epigenetic repressor, remodeling chromatin through histone monoubiquitination, *Hox*, *p16INK4a*, and master differentiation genes, and is involved in the regulation of ROS levels [42,65]. BMI1 deficiency provokes an increase in ROS mediated by mitochondrial dysfunction [93]. This crossroads between cell metabolism and progenitor cell maintenance suggests that BMI1 is pivotal for the regulation of progenitor cell identity [42,65].

In keeping with this scenario, lineage tracing of B-CPC (marked at embryonic day (E)18.5) during early postnatal transitions (P1–P14) demonstrated substantial B-CPC differentiation, correlating with an oxygen-rich postnatal environment and a net increase in ROS. No or poor expansion of labeled B-CPC was evident, especially during the P1 to P7 transition. Moreover, in accordance with the role of Bmi1 as a master regulator of redox status [94], freshly isolated B-CPC in homeostasis showed significantly lower intracellular ROS levels than cardiac Bmi1^-^ cells and cardiomyocytes, in agreement with their proposed role as cardiac progenitor cells. However, after short in vitro culture, *Bmi1* expression declines, and this phenotype can be reverted by Bmi1 lentiviral overexpression. These results suggest that BMI1 expression strongly depends on the specialized heart environment and is involved in the regulation of redox balance in B-CPC cardiac progenitors. In agreement with this interpretation, it was demonstrated that the B-CPC population is heterogeneous with regards to Bmi1 expression levels, with an inverse correlation between *Bmi1* and some cardiogenic genes (*Myh6* and *Tnnt2*). Detailed track and analysis of the B-CPC population with a population defined as cardiomyocyte-committed progenitor cells (Sca1^+^Myh6^+^ non-myocyte cells; cardioblasts), which do not express mature cardiomyocyte SαA [39], confirmed this interpretation. Accordingly, labeled cardioblasts (Myh6^MerCreMer/+^R26^Tomato/+^) were short-lived, whereas the B-CPC population remains along the whole analysis. Using a triple mouse strain, it was again demonstrated that Bmi1^+^Myh6^-^ cells (B-CPC) had the lowest levels of ROS, and Bmi1−Myh6^+^ cells showed the highest ROS levels, whereas double-positive cells (Myh6^+^Bmi1^+^) showed an intermediate ROS profile. These data strongly suggest that cardiac Bmi1^+^ cells are the origin of at least a significant portion of adult cardiomyocyte-committed cells and indicate a direct relationship between total ROS levels and cardiomyocyte commitment status. Mechanistically, a ROS-dependent Bmi1 function in B-CPC fate decisions has been established. In homeostasis, BMI1 repressed cell-fate genes, including the cardiogenic differentiation program. Oxidative damage modifies BMI1 activity in vivo, favoring antioxidant and anticlastogenic functions. Similar to the ROS-dependent epigenetic mechanism proposed for Drosophila [95], Bmi1 regulation by cell redox status has been described in B-CPC cells [42,65]. Overall, these findings demonstrate how redox status influences the cardiac progenitor responses and highlights the BMI1-mediated regulation for maintenance of cellular identity in vivo [42]. The results also strongly suggest that ROS seem to be one of the main effectors of B-CPC in differentiation [42,65].

This redox-mediated proliferation/differentiation regulatory mechanism is not restricted to stress-related situations (such as acute genotoxic damage or postnatal normoxia), as ROS-associated differentiation of cardiac progenitors in steady-state was also found. These results were further validated by comparison with G6PD^tg^ mice. Overall, these findings demonstrate how redox status influences the cardiac progenitor response and highlights redox-mediated BMI1 regulation with implications for maintenance of cellular identity in vivo [42]. Results also strongly suggest that ROS seem to be one of the main effectors of B-CPC in differentiation [42,65].

### 6.3. The Vascular Niche for B-CPC in the Adult Mouse Heart

Aiming to define the B-CPC niche, we analyzed heart sections from 5-day Tx-induced adult Bmi1-tomato mice [65]. The majority of B-CPC were located in the left ventricle, and, in all cases, B-CPC were located around the cardiac vasculature, but never in the tunica intima of blood vessels. The majority of the total B-CPC population was located within 60 μm of vasculature and preferentially close to small vessels, but with no preference to coronary veins or arteries. We verified that the observed B-CPC distribution was significantly different to the expected random distribution [65]. We verified that the observed B-CPC distribution was similar in all analyzed mice and was significantly different to the expected random distribution.

Interestingly, the spatial relationship between B-CPC and vasculature was not specific for cardiac progenitors, as a similar relationship was also found for proposed Gli1^+^ myofibroblast progenitors [96]. Of note, high levels of *Bmi1* expression are only useful for identifying B-CPC in the adult heart. In adolescent hearts, Bmi1 is expressed at significant levels in various cell types, including mature lineages, and B-CPC display an almost random distribution in adolescent hearts, suggesting that perivascular confinement becomes relevant in an age-dependent manner [65]. In addition, these results showed a decrease in B-CPC linked to heart aging.

In vivo evaluation of cell cycle status (EdU^+^) of B-CPC in relation to cardiac vasculature in a steady state demonstrated that the largest percentage of EdU^+^ B-CPC was located in a zone 40–60-μm to the nearest endothelium (50% of total B-CPC in 40–60-μm area). Whereas the largest percentage of total B-CPC resided very close to the endothelium, only a small percentage of these cells (≈10% of the total) were proliferating (20% of total B-CPC in 0−20-μm area). Equivalent analysis of infarcted hearts showed a strong distortion of the spatial distribution of B-CPC in relation to cardiac vessels, suggesting, among others, extensive cell cycle entry. Altogether, the results indicate that more quiescent B-CPC are preferentially located closer to the endothelium in comparison with the proliferating ones’ in homeostasis, indicating functional interactions. Interestingly, both scenarios of genetic manipulation of oxidative stress (reduction using the G6PD^Tg^ and vascular augmentation using *Sod3*-knockout mice) distorted the B-CPC-cardiac vessel distribution defined in steady-state conditions, again highlighting the critical role of oxidative stress levels in physiological B-CPC homeostasis and, likely, in cardiovascular pathologies. It is worth noting that the forced reduction of oxidative stress levels appears to partially release B-CPC from their confinement in the perivascular domains with very low total ROS levels, and they are able to colonize other heart domains [63]. Whether this is linked to the better preservation of B-CPC is still unknown.

Co-culture experiments of primary B-CPC with primary cardiac endothelial cells (CD31^+^) or the 1g11 endothelial cell line were compared with control primary mesenchymal cardiac cells (PDGFRα^+^).

A decrease in proliferation rate was demonstrated only in the heterotypic culture with B-CPC. In addition to cell cycle regulation, *Bmi1* expression was found to be higher in B-CPC co-cultured with endothelial cells in comparison with B-CPC cultured alone, as we expected on the basis of reported niche properties [97,98]. Endothelial cells in direct contact promoted a reduction in total ROS, which was concomitant with a net decrease in total mitochondrial mass [65], a hallmark of B-CPC in homeostasis. These results confirmed that the majority of the B-CPC are located in perivascular domains with very low total ROS levels, allowing us to propose that these structures form part of the perivascular niche for B-CPC in the adult heart.

### 6.4. Preliminary Functional Definition of the B-CPC Vascular Niche Associated Processes

On the basis of comparative RNA sequencing (RNAseq) analysis of B-CPC versus non-myocardial Bmi1^-^ adult cardiac cells, we identified vascular endothelial growth factor A/vascular endothelial growth receptor 2 (*Vegfa/Vegfr2)* and *EphrinB2 (EFNB2)/Ephrine receptor B4 (EPHB4)* as potential endothelial-related signaling pathways mediating the crosstalk with neighboring endothelial cells. VEGFA stimulation induces *Bmi1* expression in B-CPC ([65] and Albericio et al., unpublished results), similarly to how it was described in tumor cells [99]. EPHRINB2 and EPHB4 are preferentially expressed on the arterial and venous cardiac endothelium, respectively [100], binding to a fraction of B-CPC and also enhancing Bmi1 expression; this suggests that the B-CPC population comprises a mixture of artery- and venous-related perivascular cells ([65] and Albericio et al., unpublished results).

To facilitate the definition of the different processes cooperating in the B-CPC niche, we generated and characterized a conditionally immortalized cell line (B-CPC^IMM^), which maintains most of the characteristics of naïve cells (Albericio et al., unpublished results). The secretome of B-CPC^IMM^ in homeostasis was comparable with that of B-CPC, with secretomes highly enriched in pro-angiogenic factors including high levels of VEGFA, with CXCL12 (C-X-C motif chemokine ligand 12; SDF1) being the most abundant factor. Conditioned medium from B-CPC^IMM^ and B-CPC in homeostasis promoted migration of endothelial cells and angiogenesis, with both processes mediated by CXCL12. However, under oxidative stress conditions, the conditioned medium potentiated migration but inhibited angiogenesis. Finally, we found that the co-culture of 1g11 endothelial cells with B-CPC^IMM^ significantly and selectively protected against severe oxidative stress (paraquat), although the protection rates were poor when compared with other cell types such as mouse embryonic fibroblasts and the cardiomyocyte cell line HL-1.

**Figure 5:**
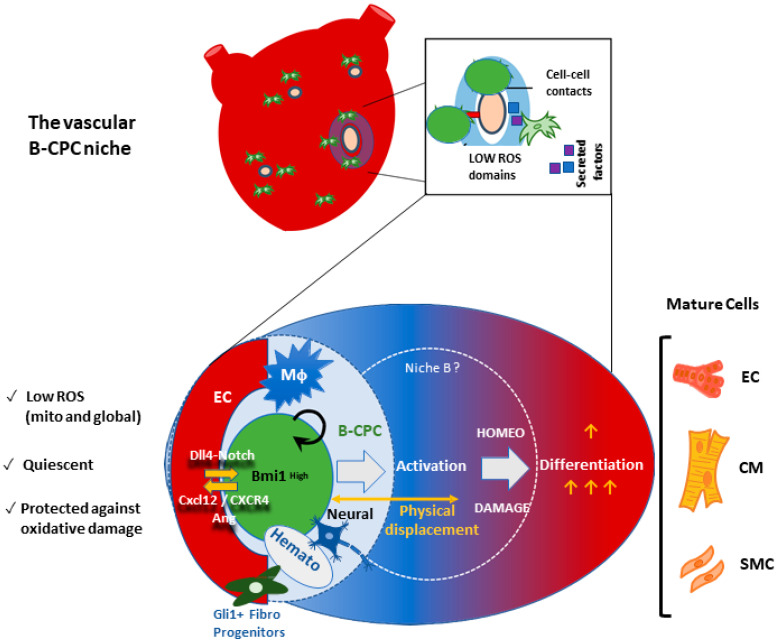
The vascular niche for cardiac B-CPC. Working model for the differentiation of B-CPC driven by ROS levels. The B-CPC are mainly confined to low-ROS perivascular spaces. They are regulated by modifications of ROS levels, cell–cell contacts, and secreted factors. B-CPC closer to the cardiac endothelium (EC), most in direct contact (B-CPC^high^), are highly preferential in quiescence, show lower levels of total and mitochondria ROS, and are more protected against oxidative stress. The demonstrated EC/B-CPC pathways (Dll4⇒Notch) and (CXCR4/angiogenesis ⇐ CXCL12) are indicated. Gli1+ fibroblastic progenitors have also been mapped in the same niches. Those cell types that are supposed to be involved in vascular niche regulation but not yet confirmed are denoted by blue symbols. MΦ, macrophages; Neural, sympathetic neural fibers; Hemato, BM-derived cells. Mature cells: cardiomyocytes (CM), smooth muscle cells (SMC) and endothelial cells (EC).

Co-culture of primary cardiac endothelial cells with B-CPC^IMM^ was demonstrated to activate the Notch pathway in the latter, likely through the expression of the Notch ligand Dll4 in the endothelial cells (Albericio et al., unpublished results; see Figure 5). EphrinB2/EphB4 have also been implicated in the regulation of the Notch pathway [101]. Indeed, the relevance of the Notch pathway is unquestioned, and its involvement in the regulation of different populations within the endothelial niche for several adult progenitors is striking [102]. For example, in the muscle satellite vascular cell niche, quiescence is regulated by *Notch*, acting in concert with *Vegf* to prevent cell senescence [103]. In good agreement with the results obtained in many adult cardiac progenitors cells, and especially in the muscle satellite vascular cell niche, it has been demonstrated that B-CPC progenitors/CSC secrete angiogenic factors and chemokines, including *Vegfa* and *Cxcl12*, promoting the structural formation of neighboring vascular tissue. Recent single-cell RNAseq analyses in mice and humans [104,105] have uncovered the complexity of the satellite muscle cell compartment and its niche, respectively.

Interestingly, adult satellite cells have been confirmed as a large heterogeneous population of stem cells and committed myogenic progenitors in skeletal muscle. At the individual level, satellite cells vary in their self-renewal, proliferation, and myogenic differentiation potential. Under conditions of regeneration, the hierarchical and collaborative behaviors of satellite cells are influenced by changes in their niche. These changes include diverse molecules, cells, and structures that constitute a highly dynamic and adaptable niche [104], enabling separation of functionally distinct satellite PAX7^+^ cell subpopulations from normal muscle [105]. This is relevant for this discussion, as the regulation of adult skeletal satellite cells seems to be the closest model to cardiac B-CPC progenitor cells.

Finally, it is important to remember that although the B-CPC population seems to be resident (not in equilibrium with an obvious extracardiac reservoir), the vascular niche could be a more permeable and interconnected structure, sensing different peripheral circulating signals and exchanging cell types more fluidly, perhaps mainly BM-derived cells. In this sense, it has been estimated that the turnover of the human cardiac endothelial lineage (15% per year) is 15-fold higher than corresponding cardiomyocytes [106], and the origin of the endothelial precursors is not completely clarified. Another plausible niche component relates to heart macrophages (if their role in the vascular niche is confirmed), which are strictly derived from BM [107]. Recently, a system of macrophages in the heart has been described that supports cardiomyocyte health by phagocytosing exopher particles ejected from cardiomyocytes that contain defective mitochondria, among other cellular contents. This macrophage population is essential to preserving metabolic stability and organ function [108]. Interestingly, another recent work in *Zebrafish* has uncovered that some macrophage populations are involved in the muscle stem cell niche, regulating their proliferation in response to injury. This transient but obligate niche (including activated macrophages) directly supplies proliferation-inducing cues that govern repair processes mediated by muscle stem cells; the main features of the model are conserved in mice [109]. Taken together, it is tempting to speculate that some specialized macrophage populations, beyond their role as scavengers for material derived from aged or damaged cardiomyocytes, could also be incorporated, transiently, into the CSC niche, in the regulation of CPC/CSC proliferation/differentiation. Indeed, phagocytosis of exopher particles by macrophages could be the activation signal for interaction with the CSC niche.

Interestingly, it has been recently demonstrated that transplantation of young BM-derived Sca-1 cells into aged lethally irradiated animals was able to reconstitute aged BM and rejuvenate the aged heart. Detailed analysis (4-month post transplantation) confirmed that the expression of senescence-associated genes in the heart was reduced, whereas rejuvenation-related genes (including Bmi1) were increased. The authors concluded that the host aged cardiac endothelial cells (GFP^-^ CD31^+^), but not cardiomyocytes, were the primary cell type rejuvenated by young Sca-1^+^ cells in a process guided by the *Cxcl12/Cxcr4* pathway [110]. These results strongly suggest that the cardiac vascular niche cells could be in a constant equilibrium and renewed by external pools of cells, mainly arising from BM.

## 7. Concluding Remarks

Ineffective ROS management is linked to the generation and progression of cardiac aging, and all suggested methods to control and delay cardiac aging focus on reducing oxidative damage. Results to date strongly suggest that the maintenance of B-CPC, a robust candidate for adult cardiac progenitor/stem cell maintenance, depends on the combinatorial regulation of several signaling pathways (dynamically orchestrated in the vascular niche). Endothelial cells play an important role in this process, and *Ephrinb2/Ephb4, Cxcl12/Cxcr4*, and *Vefga/Vegfr2* signaling are plausible mediators of the fine-tuning of endothelium–B-CPC interactions. This specialized microenvironment is confined to perivascular regions in an age-related manner, suggesting that manipulation of ROS-related pathways and/or stimulation of vascular niche-like structures would constitute an important target to mitigate cardiac injury under certain conditions.
